# Ins and Outs of Intrathecal Drug Delivery

**DOI:** 10.1002/alz70855_102613

**Published:** 2025-12-23

**Authors:** Ajay Verma

**Affiliations:** ^1^ Formation Venture Engineering, Boston, MA, USA

## Abstract

**Background:**

Intrathecal (IT) drug delivery represents a direct route to the central nervous system by bypassing the blood‐brain barrier, yet optimal drug distribution and predictable pharmacokinetics (PK) remain a challenge in clinical practice. Understanding the factors impacting the CNS entry or lymphatic clearance of different therapeutic modalities may help improve CNS therapies.

**Method:**

Using tool molecules and multi‐modal imaging techniques, including MRI, dual‐isotope SPECT/CT, cryo‐fluorescence tomography, immunohistochemistry, we investigated key in vivo determinants of IT drug disposition and distribution patterns for small molecules, oligonucleotides and nanoparticles. Studies focused on examining the impact of CSF dynamics, parenchymal influx pathways, lymphatic clearance routes and therapeutic agent molecular properties on regional CNS exposure.

**Result:**

Our findings demonstrate that IT drug distribution is governed by multiple interacting factors. Higher dosing volume correlate with rostral spread while reducing local sequestration. Molecular characteristics, particularly tissue binding affinity and size influence both parenchymal penetration and lymphatic clearance. Meningeal clearance to peripheral lymph nodes is prominent for large molecules and nanoparticles. Neuroaxial retention patterns of large molecules and nanoparticles reveals a CNS reticuloendothelial system that may play a role in elimination of certain therapeutic modalities.

**Conclusion:**

Understanding the complex interplay between dosing parameters, parenchymal influx and IT clearance routes, drug molecular properties, and patient‐specific factors may help optimize delivery to CNS regions of interest. These insights are particularly relevant given the growing interest in IT administration for treating neurological disorders, especially in conditions with altered fluid dynamics and clearance pathways such as Alzheimer's disease.

